# Exploring cell apoptosis and senescence to understand and treat cancer: an interview with Scott Lowe

**DOI:** 10.1242/dmm.023531

**Published:** 2015-11-01

**Affiliations:** Scott is principal investigator at the Memorial Sloan-Kettering Cancer Center (MSKCC) and at the Howard Hughes Medical Institute. He is also member and chair of the Cancer Biology and Genetics Program at MSKCC, chairman of the Geoffrey Beene Cancer Research Center, and professor at Weill Cornell Graduate School of Medical Sciences

## Abstract

Scott W. Lowe is currently principal investigator at the Memorial Sloan-Kettering Cancer Center. After beginning his studies in chemical engineering, he decided to take another path and became fascinated by biochemistry, genetics and molecular biology, which ultimately led to an interest in human disease, particularly cancer. During his PhD at the Massachusetts Institute of Technology (MIT), Scott had the opportunity to benefit from the exceptional mentorship of Earl Ruley, David Housman and Tyler Jacks, and contributed to elucidating how the *p53* (*TP53*) tumor suppressor gene limits oncogenic transformation and modulates the cytotoxic response to conventional chemotherapy. This important work earned him a fellowship from the Cold Spring Harbor Laboratory, which helped to launch his independent career. Scott is now a leading scientist in the cancer field and his work has helped to shed light on mechanisms of cell apoptosis and senescence to better understand and treat cancer. In this interview, he talks about this incredible scientific journey.


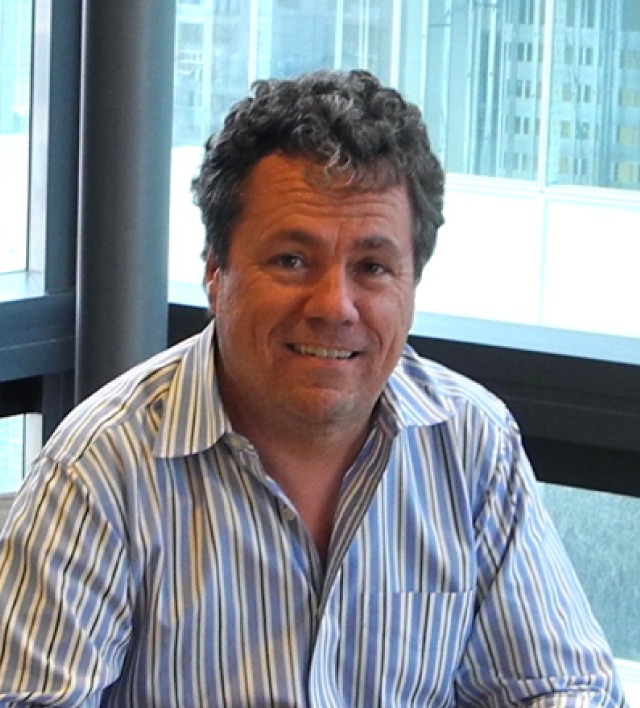


Scott W. Lowe was born in Racine, Wisconsin. He obtained his BSc degree in Biochemistry and Molecular Biology in 1986 from the University of Wisconsin-Madison, where he explored the molecular underpinnings of cholesterol metabolism and hypercholesterolemia. He then moved to the Massachusetts Institute of Technology (MIT), where he carried out his PhD project first in Earl Ruley's lab and then in David Housman's group, investigating how the adenovirus *E1A* oncogene interacted with other genes to drive malignant transformation of cells in culture. In doing so, he discovered an important role for p53 in apoptosis – a milestone discovery that opened up new avenues of research into the origin of cancer. After completing a successful year as postdoctoral fellow at MIT, Scott was awarded a fellowship from the Cold Spring Harbor Laboratory (CSHL). Here, his independent research career burgeoned and, over the years, his group has greatly contributed to understanding the role of apoptosis and senescence in cancer and how these processes affect response to treatment and drug resistance. He is now principal investigator at the Memorial Sloan-Kettering Cancer Center (MSKCC) and at the Howard Hughes Medical Institute.

**You have a Bachelor's degree in biochemistry and molecular biology. How have these studies contributed to developing your interest in cancer research?**

They had a huge impact. I started out at the University of Wisconsin-Madison as an undergraduate chemical engineer student but it just didn't fit with me. So I decided to switch focus to biochemistry and genetics. My instructors were really phenomenal and got me excited about biology, particularly genetics, biochemistry and molecular biology. I had always liked chemistry, specifically in the context of life sciences. I was fortunate enough to take an extra credit class through the biochemistry course, during which different PIs talked about their work. Through that, I met Alan Attie, who gave me the opportunity to work in his lab. I thought I would try it.

In Alan's lab, I worked initially on cholesterol metabolism and ultimately started to study the molecular basis of hypercholesterolemia in a pig strain that has a predisposition to premature atherosclerosis. Our aim was to try to understand why these animals are prone to high cholesterol. The initial hypothesis, which turned out to be only partially right, was that they had a defect in apolipoprotein B, the ligand of the LDL [low-density lipoprotein; commonly referred to as ‘the bad cholesterol’] receptor. We would isolate LDL from these pigs and perform binding assays to see whether that was true – and it was. In performing these studies I learned a lot about how ligands bind their receptors and the methods that can quantify this. To accomplish these procedures, we had to extract LDL from pigs – not an easy task – leading to many amusing stories surrounding the use and husbandry of working with large animals. The experience taught me a lot about science but also the camaraderie of working in a laboratory. It made me realize that science could be fun.

When I then went to graduate school, I actually hadn't planned to do cancer research. A lot of the work I did early on was very basic but I was excited about biology in general and, in particular, about aspects that affected human disease. I had always found placing what I was doing in the context of a human problem to be exciting, even if early on that wasn't the main driving force behind my research project.

**So it was for your PhD at MIT that you started to work on cancer?**

Yes, I did, but it's funny how this came about. As I always tell students, it often happens that when looking for a lab project one ends up working on a different problem than the one their project was initially about. This is because a project can change or take an unexpected course either because it worked or because it failed or something else came along. In my case, I joined a laboratory based on my interest in cholesterol research. The adviser that I chose, Earl Ruley, had fortuitously noticed that, in certain rat cells, some oncogenes promote lipid accumulation in a manner that is similar to what is observed in human atherosclerotic plaques. We wondered whether this could reveal an interesting link between heart disease and cancer. So, I decided to join his lab not specifically to study cancer but to work on this intriguing side project. Although the project didn't really progress – I was the only one working on it in the lab – it ended up triggering a series of amazing events that probably helped shape the rest of my career.

**You stayed at MIT for a postdoctoral position but then moved to Cold Spring Harbor Laboratory. How did this move come about?**

In Earl's lab, we made the observation that the *E1A* oncogene – an adenovirus gene that normally drives cells to proliferate – could induce *p53* [*TP53*], a tumor suppressor that acts to oppose proliferation. At the time, this was a paradoxical result. We explored how the *E1A* gene could activate p53, but, for a long time, we couldn't understand why this occurred. But about half way through my PhD, my advisor decided to leave for Vanderbilt, so I switched lab and moved to David Housman's lab. This was on the same floor of the Center for Cancer Research at MIT. My intention was to work on the Wilms’ tumor suppression gene, *WT1*, which David had just cloned. However, I returned to the project when it became clear that oncogenes could induce apoptosis – in particular from studies by Gerard Evan and Eileen White – and there were some hints that p53 might be involved. So we started to wonder whether oncogenes could drive apoptosis through p53.

In a remarkable coincidence, Tyler Jacks then started his own lab in the Center for Cancer Research (actually, in my old boss's space) and he had made a *p53* knockout mouse. As he was setting up his new lab, I started to speak with him and we decided to use his knockout mouse to explore the role of p53 in apoptosis. In the end, that resulted in a series of four papers that were very exciting at that time, showing that, through apoptosis, p53 could limit oncogenic transformation initiated by oncogenes, and could also promote cell death in response to radiation and conventional chemotherapy. I was lucky to benefit from the insight of Earl, my former thesis advisor, on oncogene cooperation. I also benefited from David Housman, who thought a lot about multidrug resistance and how cancer cells can become resistant to chemotherapy. And I benefited from Tyler: he had just started his lab, we were working with very critical reagents and he was always available, so I could talk to him every day about what was going on. So, although the disruption in the middle of my thesis work was initially stressful, in the end it all worked out and gave me the golden opportunity to interact with a lot of outstanding scientists. I was lucky also because all the papers I published had different last authors – normally the student doesn't get much credit for big advances in established labs, but I guess I did.

In any case, my work caught the attention of leaders at Cold Spring Harbor, who offered me an independent position. Again I was lucky. At Cold Spring Harbor there had been a longstanding grant, initially started by Jim Watson, awarded for studies on how DNA tumor viruses can transform cells to cancer cells. My work on E1A and apoptosis fit precisely into what they needed for an open slot on the grant.

**How did the move to Cold Spring Harbor impact on your career?**

I remember that when I was getting ready to go to Cold Spring Harbor, I had some other job offers and was initially undecided about where to go. I spoke to Phil Sharp, who was the chair of my thesis committee and who had worked at Cold Spring Harbor years ago. I asked him what I should do and he said something I thought was really profound and I have related to many students over the years. He said, “Scott, if you surround yourself with smart people everything will be all right”. I really think that's true, and was certainly true of Cold Spring Harbor. In the apoptosis field, there were Yuri Lazebnik and Michael Hengartner, and we worked together quite a lot. Eventually, I got to working extensively with Greg Hannon, which was magical. Greg is a phenomenal molecular biologist, and his skills and interests uniquely complemented my own skills in biology. Cold Spring Harbor is a great place for interacting and learning from others.

When I set up my lab, I performed simple experiments that built on what I had done as a student, which was to take mouse embryo fibroblasts, express oncogenes in them and look at whether the cells died or not. It was a simple model that required ‘turning the crank’ on a model system that we had developed and it turned out to be very powerful. People would visit CSHL and I would start talking to them about their knockout mice, and they would give me the cells so that I could test whether knockout of that particular gene affected apoptosis. I made connections that turned out to be long-lasting – with Tak Mak in particular, and with others – and it was all made possible because of the great environment at Cold Spring Harbor.

**You are also interested in cell senescence. What is the most important thing you have learned about senescence mechanisms in cancer and what needs to be investigated further?**

When we first started working on senescence, people already thought of it as a cell counting mechanism that was in place to limit the total number of times a cell can divide. This was known from the work of Hayflick and then Jerry Shay and Woody Wright and many others. We now know that that is due to telomere attrition and the activation of DNA damage responses. In studying how oncogenes promote apoptosis, we noted that the *RAS* oncogene, which is mutated in many human cancers, induced *p53* but, rather than promoting apoptosis, promoted senescence. Our work suggested that senescence might be a more broad stress response that could also protect against cancer. I think that the biggest outcome of our work in the field is that we eventually showed that senescence is an active process that limits tumorigenesis and affects therapeutic responses in tumors. We later showed that it plays a role in many physiological settings; for example, in wound repair.

“I think that the biggest outcome of our work in the field is that we eventually showed that senescence is an active process that limits tumorigenesis and affects therapeutic responses in tumors”

I think there's a lot of excitement about the potential of manipulating senescence mechanisms for therapeutic purposes, but the field suffers enormously – and this gets to the second part of your question – from the fact that the senescence process is still not very well defined. If you think back to apoptosis, we know that apoptosis at the end of the day is a process that is driven by caspases; these cleave certain proteins and you can use them as markers as they are intimately linked to the biology of the apoptotic process. For senescence, that is not the case. There are markers that we have linked to senescence but none of them are really truly specific. Maybe p16 accumulation is one, but that does not even occur in all settings that people have described as a senescence response. Still, we are increasingly appreciating the broad impact that senescence has on tissue biology. There are ways in which one may be able to modulate senescence, and that might eventually develop into therapies.

**What about manipulating the tumor microenvironment for therapeutic purposes?**

It is now clear that this is crucial and an exciting approach to therapy. In fact, immuno-oncology is one of the most promising fields in cancer therapy. I do think that we can take advantage of the ability of senescence to modulate the tumor microenvironment. Although outside the mainstream of current thinking, our work suggests that, at least in some settings, you can trigger senescence that activates the immune system to remove senescent cells. This is something we would like to provoke in a controlled way, if possible, with the ultimate aim of exploiting senescence as a therapeutic approach. Like other processes, it is likely that senescence is a beneficial response in some contexts and a detrimental one in others. That's why it is vital to be able to manipulate it in a controlled way and why more basic research is needed.

“…our work suggests that, at least in some settings, you can trigger senescence that activates the immune system to remove senescent cells”

**The use of animal models and in particular of GEMMs [genetically engineered mouse models] is key for investigating cancer mechanisms and for validating therapeutic targets. You have been using GEMMs based on embryonic stem cells; what are their advantages over other types of GEMMs?**

We can now sequence a cancer genome in 2 hours. We know a lot about the genetic changes that are associated with tumorigenesis but we don't know what it all means from a functional perspective. Deciphering all of this information is a huge challenge. GEMMs are a useful tool to investigate gene function, as they can mimic cancer-related mutations and provide a model system in which to study tumorigenesis in the setting of a functioning tissue microenvironment. The problem is that GEMMs are ridiculously slow and expensive to produce, especially in the context of cancer, as you have to intercross multiple mutants to get a configuration that can replicate the complexity of the human tumor. With all the sequencing that has been done, we also know that there are hundreds of configurations that occur in human tumors, which you will never be able to model using standard intercrossing. In GEMMs that are based on embryonic stem [ES] cells, we try to introduce multiple oncogenic lesions directly into the ES cells, which allows for the production of mice already harboring multiple genetic changes that contribute to cancer, without all the crossing. The idea is to capture the advantages of GEMMs but to reduce the time that it takes to produce them and also to increase the possibilities of experimental manipulations.

“GEMMs are a useful tool to investigate gene function, as they can mimic cancer-related mutations and provide a model system in which to study tumorigenesis in the setting of a functioning tissue microenvironment”

One of the things that we have incorporated into these models is the ability to introduce a conditional short-hairpin RNA that targets a gene of interest. Taking advantage of RNAi's ability to suppress gene expression, this approach allows us to inactivate or reactivate a gene at different stages of tumorigenesis to provide not only an assessment of how tumors develop or initiate but also of what is required to sustain them once they have actually been produced. This is important in the context of validating therapeutic targets. The requirement for gene function at the beginning of cancer development may be quite different than that of later stages. In most cases, we deal with patients who already have a tumor and so we need to know what a gene is doing in that context, so that we can move towards personalizing treatment.

This work is continually evolving, and we almost have a pipeline in which you can engineer a mouse model to relatively rapidly assess whether a particular gene is contributing to cancer initiation or whether it helps maintain that cancer, and then use the same system as a preclinical model to validate the pathway as a therapeutic approach. Of course, we have to keep in mind that there are differences between animal models and patients, and, as we move into clinical trials, these differences can become apparent.

**What are the key challenges involved in developing new anti-cancer drugs, and how do you tackle them?**

I think I am still in a learning phase. One of the reasons I moved to the Memorial Sloan-Kettering Cancer Center is to be in a position in which the work we do could ultimately be translated into patient therapies. We have had success doing this in terms of laying the foundations for clinical trials. And this is where I have learned new lessons; one is that it isn't enough to just have a good idea – someone has to pay for the idea to be tested and eventually brought to fruition. That requires in most cases the buy-in from the pharmaceutical companies that are producing the drugs. Several aspects related to developing a clinical trial, including the financial aspects, were certainly new to me. Also, I am learning that there are many factors that will determine the success or failure of a promising target – even when it is very well validated in the preclinical phase – including the chemical nature of the compound used to modulate the target, whether the compound has off-target effects, whether there is a tissue that is totally irrelevant to the tumor in which a target gene might exert an important function. These broad issues have encouraged us to use mouse models not only to validate targets but also to try to understand toxicities: for example, if we inhibit a target systemically in a mouse, are there any responses arising to predict what might go wrong in humans? These challenges are what everybody in the field faces.

“I am learning that there are many factors that will determine the success or failure of a promising target – even when it is very well validated in the preclinical phase”

**Along with your research appointments you also have academic duties. Are you able to still spend time in the lab? How can a group leader find the right balance between research and teaching?**

It is easy to get caught up in other responsibilities for which there is often a deadline, and a meeting with your postdoc can get pushed back very easily. So one of the things I feel is most important for a lab head is to encourage people to be really interactive, to be talking to each other and exchanging ideas. During my training at MIT, I didn't talk to my boss every day but I was constantly running ideas by my colleagues and trying to learn new techniques. I think that encouraging an interactive and supportive environment helps with the fact that I can't be in the lab as much as I would like. I don't have many teaching responsibilities in the didactic sense, which does help – I love to do it but it does take a lot of time. Obviously I have teaching responsibilities with graduate students and with developing their thesis work.

“…research discoveries generate public issues that need to be addressed – some of them are ethical, some medical, others related to privacy – and better educating and involving the public is really important for society to make the right choices”

**What do you think about academic teaching? Is it a kind of moral obligation for a scientist to transfer knowledge to the next generation or is it something the teachers themselves benefit from?**

I think it's both. My career was influenced by some great teachers that stimulated me to pursue a career in research. But the process of teaching also stimulates development of your own ideas. If I teach a lecture on some aspects of cancer biology that I haven't thought about for a while, sometimes I realize these might be related to the work being done in the lab. To take it a step further, although it is very important to educate students – people who have committed to learning science – I also believe that it's also important to educate the public, who supports most of the work that we do and thus deserves to know what their investment is accomplishing. This can help create enthusiasm to support basic research, without over-representing what its short-term impact on human health might be. For example, if there has been a successful clinical trial for an anti-cancer drug, this is obviously something that the public is very interested in. It is harder to get people excited about discovering how p53 works at the molecular level, although this information is very important for understanding and eventually treating cancer. In addition, research discoveries generate public issues that need to be addressed – some of them are ethical, some medical, others related to privacy – and better educating and involving the public is really important for society to make the right choices. I think this is an important aspect of what scientists do and also an obligation that we have.

**One last question not related to research. How do you relax away from the lab?**

Whenever I can, I go camping, hiking and backpacking. I love to ski – I go a couple of times a year. I also like to get outside and wander around town; New York City is a great place to hang out in.

